# Editorial: Veins in the brain

**DOI:** 10.3389/fsurg.2023.1269196

**Published:** 2023-08-22

**Authors:** Emanuela Crobeddu, Edoardo Agosti, Christian Cossandi, Valentina Tardivo, Lucio De Maria, Simona Serioli, Marco Maria Fontanella, Luca Valvassori

**Affiliations:** ^1^ Section of Neurosurgery, Department of Neuroscience, University of Turin, Turin, Italy; ^2^ Department of Neurosurgery, Maggiore Della Carità University Hospital, Novara, Italy; ^3^ Division of Neurosurgery, Department of Medical and Surgical Specialties, Radiological Sciences and Public Health, University of Brescia, Brescia, Italy; ^4^ Department of Neurosurgery, ASST Santi Paolo e Carlo, Milano, Italy; ^5^ Department of Neuroradiology, ASST Santi Paolo e Carlo, Milano, Italy

**Keywords:** cerebral veins, cerebrovascular malformations, etiopathogenesis, hemorrhagic risk, aterovenous malformations, aterovenous fistulas, cavernomas

**Editorial on the Research Topic** Veins in the brain

The comparison between “veins in the brain” and “clouds in the sky” illustrates a significant analogy. Just as large clouds can predict an impending storm or the absence of clouds may lead one to believe that rain won't come, cerebral veins play a crucial role in the pathophysiology, clinical presentation, and prognosis of several cerebrovascular malformations (CVMs) (1, 2). This issue is dedicated to analyzing the role of cerebral veins in conditioning CVMs clinical and treatment outcomes. The evolving understanding of cerebral veins dynamic nature has shed light on their contributions to the pathogenesis, clinical manifestation, and prognosis of CVMs. Further research on cerebral veins promises to improve our ability to predict and manage CVMs effectively.

Historically, cerebral veins were of limited interest in the scientific community. In recent decades, compelling evidence has emerged highlighting their active and pivotal role in the pathogenesis of CVMs (Chen et al.) (3). Accordingly, cerebral veins are no longer sees as passive observers; instead, they are recognized as dynamic characters in the genesis and progression of CVMs (Agosti et al.) (4, 5). For instance, it is now well-established that brain arteriovenous malformations (bAVMs) with deep venous drainage or the presence of a solitary superficial main draining vein, instead of multiple drainages, are more prone to bleeding (Zhang et al.). Moreover, the importance of cerebral vein pathophysiology is further underscored by the devastating outcomes associated with cerebral sinus thrombosis, even in young patients. Likewise, dural arteriovenous fistulas (AVFs) can cause massive bleeding, often triggered by thrombosis of the venous segment (Agosti et al., Vercelli et al.).

Numerous CVMs have been shown to exhibit associations, such as cavernomas and developmental venous anomalies (DVAs), dural AVFs and DVAs, or dAVFs and bAVMs, among others (Agosti et al.) (6, 7). Often, the coexistence of these CVMs is linked to a worse clinical outcome, and it has been postulated that cerebral veins may serve as a shared promoter in the etiopathogenesis of these lesions (Agosti et al.) (8). For example, DVAs are typically regarded as non-pathological lesions; however, their association with arteriovenous shunts appears to carry a more adverse clinical connotation, increasing the propensity for bleeding incidents (9). In this issue, we provide a comprehensive description of the association between DVAs and dAVFs, discuss the hemorrhagic risk, and postulate an etiopathogenetic theory on *de novo* formation of DVAs (Agosti et al.). Moreover, while a substantial body of literature exists concerning the treatment and prognosis of brain AVFs, our understanding of spinal AVFs and the role of surgical intervention remains comparatively limited (Vercelli et al.). For this reason, this issue aims to better analyzed the recurrence rate after surgical and endovascular treatment of spinal dural and epidural AVFs.

Brain AVMs represent rare vascular diseases, and their pathogenesis remains a subject of controversy. Traditionally considered congenital malformations, mounting evidence has shown that these AVMs can manifest or regress over the course of an individual's lifetime (8, 10, 11). In this issue, we delve into the molecular and genetic mechanisms that underlie bAVMs pathogenesis, shedding light on the multifactorial origin of these malformations. Additionally, we thoroughly investigate the factors that influence bAVM-related hemorrhage and critically evaluate the methodological rigor of existing genetic studies pertaining to bAVM-related hemorrhage (12). The comprehensive exploration of the molecular mechanisms and risk factors associated with bAVMs is poised to advance our understanding of these intricate vascular anomalies, ultimately contributing to improve management and therapeutic strategies. Notably, the incidence of incidentally detected bAVMs has raised in parallel with the increased utilization of neuroradiological examinations. Consequently, there is considerable interest in developing a predictive model for rupture risk, and three-dimensional morphological features extracted from computed tomography angiography have emerged as a promising and valuable approach for this purpose (Zhang et al.).

Several aspects of CVMs involving cerebral veins continue to elude us. However, significant progress in comprehending cerebral veins dynamic nature and role in triggering CVMs formation and growth is being made. The once-clouded sky of uncertainty is gradually clearing, affording us a clearer perspective of the path ahead. With ongoing research and a deeper grasp of the intricate relationship between cerebral veins and CVMs, we strive towards further advancements in the diagnosis, management, and ultimately the outcomes of patients affected by these complex vascular lesions.
